# Echocardiographic Findings in Patients with Atrial Septal Aneurysm: A Prospective Case-Control Study

**DOI:** 10.1155/2019/3215765

**Published:** 2019-04-01

**Authors:** Ramazan Atak, Mehmet Ileri, Selcuk Ozturk, Ahmet Korkmaz, Ertan Yetkin

**Affiliations:** ^1^Lokman Hekim Akay Hospital, Department of Cardiology, Ankara, Turkey; ^2^Ankara Numune Education and Research Hospital, Department of Cardiology, Ankara, Turkey; ^3^Ankara Education and Research Hospital, Department of Cardiology, Ankara, Turkey; ^4^Istinye University Liv Hospital, Department of Cardiology, Istanbul, Turkey

## Abstract

**Background:**

Atrial septal aneurysm (ASA) is a congenital deformity of the interatrial septum with a prevalence of 1-2% in the adult population. Although ASA has been supposed to be an incidental finding in echocardiographic examination, its structural and clinical associations have gained an increasing interest.

**Aim:**

To investigate and compare the clinical features and echocardiographic parameters between ASA patients and age- and gender-matched control group patients.

**Methods:**

410 patients with ASA were enrolled in the study, prospectively. After the exclusion of 33 patients, the remaining 377 patients comprised the study group. The control group consisted of 377 age- and gender-matched patients without ASA.

**Results:**

Aortic valve regurgitation and mitral valve regurgitation were more often observed in patients with ASA, and percentages of patients with ascending aortic aneurysm (AAA), patent foramen ovale (PFO), and atrial septal defect (ASD) were higher in ASA patients compared to control group patients. Aortic root diameter was larger in ASA patients compared to control group patients (29.2 ± 3.9, 28.6 ± 3.1, *p*=0.05, respectively). Ascending aorta diameter was higher in ASA patients compared to patients without ASA (44 ± 0.3, 41.5 ± 0.2, *p*=0.02). Logistic regression analysis revealed that mitral valve regurgitation (OR: 2.05, 95% CI : 1.44–2.92, *p* < 0.001) and PFO (OR: 11.62, 95% CI : 2.64–51.02, *p*=0.001) were positively and independently associated with the presence of ASA. AAA tended to be statistically and independently associated with ASA (OR: 2.69, 95% CI : 0.97–7.47, *p*=0.05).

**Conclusions:**

We have demonstrated a higher incidence of mitral/aortic valvular regurgitations, AAA, PFO, and ASD in ASA patients compared to age- and gender-matched control group patients. In addition, we have shown that ASA is significantly and positively associated with mild mitral regurgitation and PFO.

## 1. Introduction

Atrial septal aneurysm (ASA) is a congenital deformity of interatrial septum consisting redundant and mobile interatrial septal tissue in the region of fossa ovalis with bulging into the right or left atrium and sometimes oscillating between both atria. It is a rare and incidental finding in the adult population [1, 2]. The prevalence of ASA is 2-3% in adult population [3]. The clinical relevance of this cardiac anomaly is uncertain, but several reports suggest a potential role in cardioembolic stroke [3–5]. It is usually related with other cardiac abnormalities such as mitral valve prolapse (MVP) [6], patent foramen ovale (PFO), and atrial septal defect (ASD) [7].

Recently, Yetkin et al. investigated the prevalence and characteristics of ASA in a relatively large patient population including 16570 adult patients undergoing transthoracic echocardiography (TTE). Prevalence of ASA was documented in 2.4% of patients with a female dominance (72%). Additionally, they demonstrated valvular regurgitations, and supraventricular arrhythmias are the most often accompanying pathologies to ASA [2]. In another study including 15234 patients recently published by Jatav et al., similar findings were observed in terms of prevalence and gender [8]. In the present study, we hypothesized that echocardiographic abnormalities including valvular pathologies and congenital heart defects are more common in ASA patients. Therefore, we aimed to compare the clinical characteristics and echocardiographic parameters between ASA patients and age- and gender-matched control group patients.

## 2. Methods

### 2.1. Study Population

**Figure 1:**
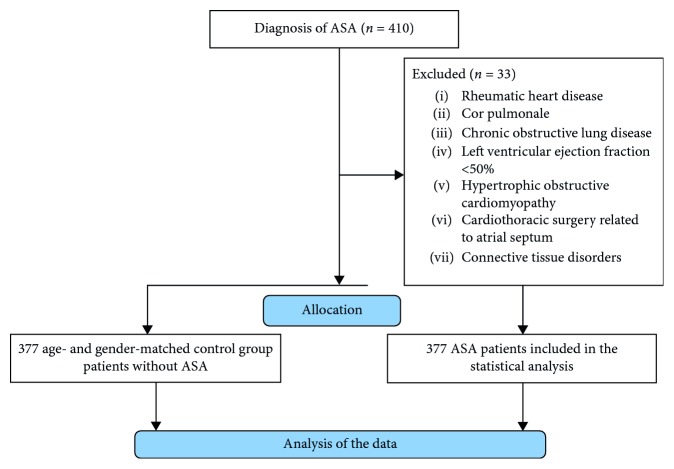
Flowchart of the study. ASA: atrial septal aneurysm.

The local ethics committee of Ankara Numune Education and Research Hospital approved the study protocol, and the study was conducted according to the Helsinki Declaration. Written informed consent was obtained from the study participants. 410 patients with ASA were prospectively enrolled in the study in a consecutive manner. Patients older than 18 years old were recruited from the outpatient cardiology clinic between January 2016 and February 2018. The main complaints of patients were chest pain (46%), dyspnea (37%), palpitation (35%), fatigue (9%), venous disease leg symptoms (9%), sweating, nausea, and vomiting (5%), and syncope (2%). After the exclusion of 33 patients who did not match the inclusion criteria, the remaining 377 patients comprised the study group and were included in the statistical analysis. The control group consisted of 377 patients who fulfilled the inclusion criteria without ASA and were age- and gender-matched with ASA patients. The control group patients were included in the study prospectively. Patients with rheumatic heart disease, cor pulmonale, chronic obstructive lung disease, left ventricular ejection fraction <50%, hypertrophic obstructive cardiomyopathy, and cardiothoracic surgery involving atrial septum were excluded from the analysis. Connective tissue disorders such as rheumatoid arthritis, systemic lupus erythematosis, scleroderma, and Marfan syndrome were also regarded as exclusion criteria. However, there were no patients excluded because of connective tissue disease in both of the groups. Patients' history of connective tissue disease, examination, and laboratory findings suggestive of connective tissue disorders guided the exclusion of patients. The flowchart of the study including exclusion criteria is also shown in Figure 1.

Patients who were included in the study were examined for baseline clinical parameters and recorded on a study chart. Arterial hypertension was defined as repeated blood pressure measurements ≥140/90 mm·Hg or usage of antihypertensive drugs. Diabetes mellitus (DM) was defined as fasting plasma glucose levels more than 126 mg/dL in multiple measurements or glucose level over 200 mg/dL at any measurement or active use of antidiabetic medications. Smoking was defined as current smoking in the previous six months. Hyperlipidemia was defined as a baseline cholesterol level of >200 mg/dl and/or a low-density lipoprotein cholesterol level of >130 mg/dl or previously diagnosed and treated hypercholesterolemia. Coronary artery disease (CAD) was defined as electrocardiographic signs of prior myocardial infarction, echocardiographic findings of left ventricular wall motion abnormalities indicating myocardial infarction, and angiographic proven severe coronary artery stenosis >50%. Ischemic stroke was defined as a new-onset neurological deficit of vascular origin diagnosed by a neurologist lasting 24 hours or longer or until death without evidence of primary intracranial bleeding.

### 2.2. Transthoracic Echocardiography

**Figure 2:**
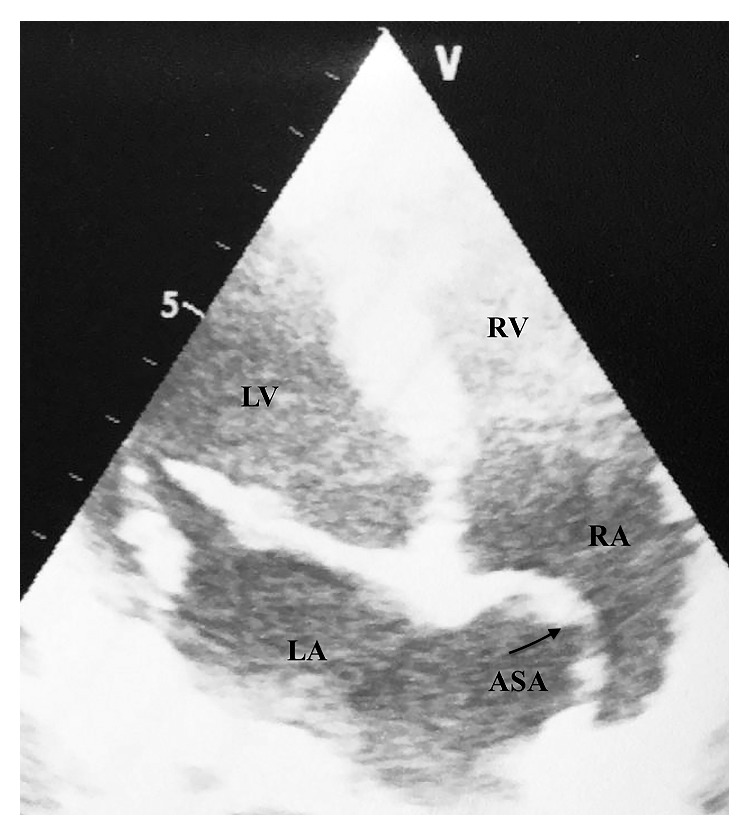
Apical four chamber view of atrial septal aneurysm detected by transthoracic echocardiography. ASA: atrial septal aneurysm, LV: left ventricle, LA: left atrium, RV: right ventricle, and RA: right atrium.

All of the TTE examinations were performed with the use of VividS5 Pro System (General Electric Medical Systems, Milwaukee, Wisconsin, USA) with a 2.5 MHz phase array during the outpatient cardiology clinic admission of participants. The same cardiologist, who was blinded to the study protocol, did cardiovascular examinations and echocardiographic measurements during the enrollment period. Patients underwent standard imaging measurements including left atrial, aortic annulus, ascending aorta, left ventricular end diastolic and systolic diameters at left lateral decubitus position including parasternal long and short axis, and apical four and five chamber views. If needed, additional measurements were done from the suprasternal and subcostal views. All echocardiographic procedures were compatible with the recommendations of the American Society of Echocardiography [9]. Aortic valve regurgitation severity and mitral valve regurgitation severity were measured by the recommendations of the European Association of Echocardiography. Briefly, small aortic regurgitation jet width was graded as mild, intermediate jet as moderate and large in central jet, and variable in eccentric jets as severe aortic regurgitation. Likewise, small mitral central color flow jet was graded as mild, intermediate jet as moderate, and very large central jet or eccentric jet adhering, swirling, and reaching the posterior wall of the left atrium as severe mitral regurgitation [10]. ASA was defined as according to the criteria previously published by Hanley and colleagues [11]. In summary, ASA was considered to be present if protrusion of interatrial septum was more than 15 mm into the left or right atrium or phasic excursion more than 15 mm during the respiratory cycle, and the base of the aneurysm was at least 15 mm in diameter. PFO was defined as the observation of right to left interatrial shunt diagnosed by color Doppler imaging and/or intravenous injection of agitated saline at rest or with provocative maneuvers such as cough or valsalva. Ascending aorta aneurysm (AAA) was diagnosed as if ascending aortic diameter was equal or over the 4.0 cm [2, 12, 13]. An image of ASA diagnosed by TTE is represented in Figure 2.

Transesophageal echocardiography (TEE) examination was performed only in selected patients. TEE was performed to rule out the presence of PFO, to assess ASD in case of suspicion, or to make definitive diagnosis. Assessment of ASA was performed only by TTE. Briefly, the patient was anesthetized with a topical agent (lidocaine) at least after a 4-hour fasting state, and esophageal intubation was performed at left lateral decubitus position. As mentioned above, TEE examination was not applied to all patients. Diagnosis of PFO and ASD was ascertained by both color Doppler imaging and intravenous injection of agitated saline at rest and during valsalva maneuver during TEE examination. Projections and measurements were done according to the guideline of American Society of Echocardiography [9]. Among the whole study group including 754 patients, only 42 patients applied TEE examination.

### 2.3. Statistical Analysis

All statistical analyses were performed using SPSS version 16.0 (SPSS, Inc., Chicago, Illinois). Continuous variables were expressed as mean ± standard deviation, and categorical variables were presented as counts and/or percentages. Comparison of parametric values between the two groups was performed by means of independent samples *t* test. Categorical variables were compared by the chi-square test. Logistic regression analysis was performed to identify the possible associates of ASA with echocardiographic parameters, namely, left ventricular, left atrial and aortic root diameters, mitral and aortic valve regurgitations, AAA, mitral valve prolapse, PFO, ASD, and ventricular septal defect (VSD). A two-tailed *p* < 0.05 was considered as statistically significant.

## 3. Results

Baseline clinical, demographic characteristics, echocardiographic measurements, and pathologies of the study population are presented in Table 1. There were no difference between the groups in respect to age, gender, hypertension, DM, smoking status, CAD, and hyperlipidemia. Echocardiographic parameters including left atrium anterior-posterior dimension, left ventricle end diastolic and systolic diameter, ejection fraction, MVP, and presence of VSD were similar between the groups. Aortic root diameter was larger in ASA patients compared to control group patients (29.2 ± 3.9, 28.6 ± 3.1, *p*=0.05, respectively). Ascending aorta diameter was higher in ASA patients compared to patients without ASA (44 ± 0.3, 41.5 ± 0.2, *p*=0.02). Aortic valve regurgitation (16.1% vs. 11.1%, *p*=0.04, respectively) and mitral valve regurgitation (39.2% vs. 24.6%, *p* < 0.001, respectively) were more often observed in patients with ASA than control group patients. Among those 148 patients with mitral regurgitation in the ASA group, 145 patients (98%) had mild mitral regurgitation in severity. In addition, percentages of patients with AAA (4.5% vs. 1.5%, *p* < 0.001, respectively), PFO (6.1% vs. 0.5%, *p* < 0.001, respectively), and ASD (2.3% vs. 0.2%, *p*=0.01, respectively) were significantly higher in ASA patients than those of control group patients.

Logistic regression analysis revealed that mitral valve regurgitation (OR: 2.05, 95% confidence interval: 1.44–2.92, *p* < 0.001) and PFO (OR: 11.62, 95% confidence interval: 2.64–51.02, *p*=0.001) were positively and independently associated with the presence of ASA, as shown in Table 2. AAA tended to be statistically and independently associated with ASA (OR: 2.69, 95% CI : 0.97–7.47, *p*=0.05). Other echocardiographic measurements including left anterior-posterior dimension, left ventricle end diastolic and systolic diameter, aortic root diameter, and pathologies including aortic valve regurgitation, MVP, ASD, and VSD were not associated with ASA.

## 4. Discussion

Interatrial septum has three components named as septum primum, septum secundum, and atrioventricular canal septum. At different embryological stages during gestation, these three components fuse and form “fossa ovalis,” which allows for the continuous passage of oxygenated placental blood from the right atrium to left portion of the heart. Physiologically, interatrial septum permits this shunt until birth, and after birth, it is closed with the increase in pulmonary blood flow. However, after birth, there may be certain locations where interatrial septum is defective causing PFO, ASD, or ASA [14–16].

Although the first report of ASA was published in 1934 by Lang and Posselt [17], it has gained special interest since the demonstration of its association with systemic embolism in previous reports [11, 18–21]. It was defined as a rare congenital abnormality initially but with the development and widespread use of two-dimensional TTE, its determinability has increased [6]. Its prevalence was reported to vary between 2 and 3% in previous echocardiography studies with a female dominance [2, 3, 8]. However, it should be noted that the prevalence and gender dominance may be affected by several factors depending on the patient population, accepted diagnostic criteria, and applied diagnostic modality. There are several cut-off levels used in different studies for diagnostic criteria of ASA varying between 6 and 15 mm [11, 22, 23]. In our study, we applied the diagnostic criteria defined by Hanley et al. in 1985 [11], which we have briefly described above. Due to its widespread applicability and accessibility, TTE is accepted as the main diagnostic modality in ASA patients. However, TEE was suggested to be superior to transthoracic examination for detecting interatrial septum abnormalities such as PFO and ASA [22, 24]. In our study, we examined our patients with TTE. We did not apply TEE to all of the patients. In case of suspicion or measuring the size of ASD, we performed TEE only in selected patients.

ASA was shown to be linked with congenital heart diseases including mostly PFO [25] and ASD [22, 23], which are also known to be associated with systemic embolism. In addition, valvular pathologies such as MVP [6, 11, 26], aortic and mitral valve regurgitation [2, 8], and AAA [2] are known to be frequent in these patients. Cabanes et al. investigated the role of ASA in partially young cryptogenic stroke patients and found that ASA and PFO are both significantly linked with stroke compared to control group patients. In their study, they applied TEE to all of the patients [25]. Mügge et al. investigated TEE applied ASA patients, retrospectively. They showed that TEE is superior to TTE for the detection of ASA, and ASA is a risk factor for cardiogenic embolism. In addition, they found that the most common abnormalities associated with ASA are congenital heart defects, especially PFO [22]. Compared to previous studies, we found similar results in terms of congenital heart diseases and stroke. PFO and ASD were more frequent in ASA patients; however, only PFO showed significant association with ASA in logistic regression analysis. VSD was not different among the groups. In a recently published study by Yetkin et al. including 16570 patients referred for TTE, mitral and aortic valve regurgitations were the most common pathologies in ASA patients. In their study, 39% of ASA patients had mitral regurgitation and 16% of patients had aortic regurgitation [2]. In our study, valvular pathologies including aortic and mitral valve regurgitation were significantly more frequent in ASA patients. In addition, AAA was more frequent in ASA patients, and ascending aortic root diameter was significantly higher in ASA patients. Nevertheless, in logistic regression analysis, only mitral valve regurgitation was significantly and independently associated with ASA. Accordingly, high frequency of valvular regurgitations and AAAs in ASA patients compared to their counterparts underlies the possible role of connective tissue pathology in the pathophysiology of ASA [27]. Consistent with this hypothesis, increased prevalence of ASA was documented and discussed in patients suffering from Behçet's disease, which is a systemic vasculitis involving extracellular tissue similar to AAA in previous reports [27–29].

Mitral valve regurgitation develops when the mitral valve leaflets do not sufficiently cover the mitral annular orifice throughout LV systole, and in the absence of left ventricular wall and myocardial functional abnormality, it is commonly classified as primary mitral regurgitation. Primary mitral regurgitation refers to abnormal function of mitral leaflets, commissures, and chordae itself. Although it is not in the scope of this study, slight weakness of the extracellular matrix of the valvar structure might have resulted in mitral regurgitation due to changes in mitral leaflet pliability and motion. Given the fact that increased blood pressure correlates with higher left ventricular pressure, and this, in turn, exposes the mitral and aortic valve to higher physical stress. Long-term exposure to higher blood pressure may also lead to structural and functional changes of the mitral valve and aortic valve resulting in regurgitation [30, 31]. However, similar rates of hypertension in patients with and without ASA eliminate the role of hypertension as a contributing factor for valvular regurgitation in our study population. In addition, cardiovascular risk factors such as DM, smoking, hyperlipidemia, and CAD itself did not differ significantly between patients with and without ASA. Factors, which may directly affect left or right atrial pressure differences such as left ventricular ejection fractions and left atrial and ventricular dimensions, were also comparable between groups.

MVP has been supposed to be another accompanying pathology of ASA [6, 11, 32]. It was hypothesized that the linkage between two diseases is the connective disease of the fibrous tissue of the heart [6, 32]. However, we did not observe any difference regarding the presence of MVP between the two groups. We have documented very low prevalence of MVP in both patient groups, which may be explained by more strict diagnostic criteria of MVP [33]. Consistent with our findings, Jatav et al. have also found very low percentage of MVP in their ASA patients [8]. Echocardiographic prevalence of MVP has decreased significantly in recent decades due to the changes in echocardiographic diagnosis criteria [34]. It is difficult to make a definite causality between the ASA and connective tissue disease in the light of this study. Although connective tissue disease was defined as exclusion criteria, regarding the coexistence and association of AAA, ASA, and mitral regurgitation which occur due to structural changes in the connective tissue, it is possible to exist a causal link between ASA and other associates through changes in the connective tissue structure rather than a definite connective disease itself.

The present study has a few limitations. Since all patients both in the ASA group and control group were enrolled to the study from outpatient cardiology clinic, these results may not be generalized to whole population. Second, it would be better if we had evaluated the ASA with TEE in all cases, which was demonstrated to be superior to TTE [22]. However, due to the study design, we applied TEE only in selected patients when needed. This might have resulted in underestimation of PFO in both ASA and control groups. On the other hand, statistically significant high rate of PFO compared to control subjects still validates the significant association of PFO with ASA.

In conclusion, we have demonstrated higher percentage of mitral/aortic valvular regurgitations, AAA, PFO, and ASD in ASA patients compared to age- and gender-matched control group patients. In addition, we have shown that ASA is significantly and positively associated with mild mitral regurgitation and PFO and tended to be associated with AAA. Further clinical and preclinical studies are warranted to elucidate the pathophysiological associates or contributors of ASA.

## Figures and Tables

**Table 1:** Baseline demographics, echocardiographic parameters, and pathologies of the study population.

Variable	Atrial septal aneurysm		*p* value
	No (*n*=377)	Yes (*n*=377)	
Age (years)	49 ± 15	49 ± 15	0.86
Women (*n*)	242 (64.2%)	242 (64.2%)	1.00
Hypertension (*n*)	113 (29.9%)	116 (30.7%)	0.81
Diabetes mellitus (*n*)	46 (12.2%)	44 (11.6%)	0.82
Current smoker (*n*)	66 (17.5%)	56 (14.8%)	0.37
Coronary artery disease (*n*)	64 (16.9%)	71 (18.8%)	0.50
*Ischemic stroke (n)*	**3 (0.8%)**	**14 (3.7%)**	**<0.001**
Hyperlipidemia (*n*)	60 (15.9%)	64 (16.9%)	0.69
Left atrium anterior-posterior dimension (mm)	37.0 ± 4.8	36.9 ± 4.8	0.64
Left ventricle end diastolic diameter (mm)	45.6 ± 3.8	45.7 ± 3.9	0.70
Left ventricle end systolic diameter (mm)	28.4 ± 2.9	28.5 ± 2.8	0.60
Aortic root diameter (mm)	28.6 ± 3.1	29.2 ± 3.9	0.05
*Ascending aorta diameter (mm)*	**41.5** **±** **0.2**	**44** **±** **0.3**	**0.02**
Ejection fraction (%)	67.4 ± 2.5	67.1 ± 3.0	0.08
*Aortic valve regurgitation (n)*	**42 (11.1%)**	**61 (16.1%)**	**0.04**
Mild	36 (85.7%)	50 (82.0%)	
Moderate	6 (14.3%)	10 (16.4%)	
Severe	0 (0%)	1 (1.6%)	
*Mitral valve regurgitation (n)*	**93 (24.6%)**	**148 (39.2%)**	**<0.001**
Mild	89 (95.7%)	145 (98.0%)	
Moderate	4 (4.3%)	2 (1.35%)	
Severe	0 (0%)	1 (0.67%)	
*Ascending aortic aneurysm*	**6 (1.5%)**	**17 (4.5%)**	**<0.001**
Mitral valve prolapse	2 (0.5%)	3 (0.7%)	0.72
*Patent foramen ovale (n)*	**2 (0.5%)**	**23 (6.1%)**	**<0.001**
*Atrial septal defect (n)*	**1 (0.2%)**	**9 (2.3%)**	**0.01**
Ventricular septal defect (*n*)	1 (0.2%)	1 (0.2%)	1.00

**Table 2:** Logistic regression analysis of the echocardiographic parameters and pathologies associated with atrial septal aneurysm.

Variable	*p* value	Odds ratio	95% confidence interval
Left atrium anterior-posterior dimension	0.29	0.81	0.55–1.19
Left ventricle end diastolic diameter	0.67	0.87	0.47–1.61
Left ventricle end systolic diameter	0.97	1.01	0.44–2.34
Aortic root diameter	0.25	1.35	0.80–2.26
Aortic valve regurgitation	0.73	1.08	0.66–1.77
*Mitral valve regurgitation*	**<0.001**	**2.05**	**1.44–2.92**
Ascending aortic aneurysm	0.05	2.69	0.97–7.47
Mitral valve prolapse	0.86	0.84	0.11–6.41
*Patent foramen ovale*	**0.001**	**11.62**	**2.64–51.02**
Atrial septal defect	0.23	3.88	0.41–36.08
Ventricular septal defect	0.77	1.51	0.09–24.59

## Data Availability

The data used to support the findings of this study are available from the corresponding author upon request.
